# Disodium dihydrogen pyridine-2,3,5,6-tetra­carboxyl­ate trihydrate

**DOI:** 10.1107/S1600536810012870

**Published:** 2010-04-14

**Authors:** Fang-Hong Hu, Jian-Li Lin

**Affiliations:** a State Key Laboratory Base of Novel Functional Materials and Preparation Science, Center of Applied Solid State Chemistry Research, Ningbo University, Ningbo, Zhejiang 315211, People’s Republic of China

## Abstract

In the title compound, 2Na^+^·C_9_H_3_NO_8_^2−^·3H_2_O, the asymmetric unit consists of two Na^+^ cations, one dihydrogen pyridine-2,3,5,6-tetra­carboxyl­ate dianion (H_2_pdtc^2−^) and three water mol­ecules coordinated to the Na^+^ cations. The configuration of the anion is stabilized by intramolecular O—H⋯O hydrogen bonding between vicinal carboxylate/carboxy groups. The Na^+^ cations are bridged by the H_2_pdtc^2−^ dianions, generating layers extending infinitely in sheets parallel to (001), and further pillared by the water mol­ecule linkers to build up a three-dimensional framework.

## Related literature

For related compounds involving the pyridine-2,3,5,6-tetra­carboxylic acid ligand, see: Zhang *et al.* (2010 ▶); Yang *et al.* (2008 ▶); Sun, Zhou & An (2009 ▶); Sun, Zhou & Yan (2009 ▶).
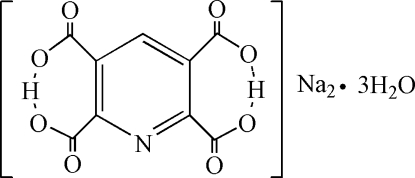


## Experimental

### 

#### Crystal data

2Na^+^·C_9_H_3_NO_8_^2−^·3H_2_O
*M* *_r_* = 353.15
Triclinic, 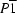

*a* = 5.5844 (11) Å
*b* = 6.6770 (13) Å
*c* = 18.631 (4) Å
α = 81.34 (3)°
β = 86.77 (3)°
γ = 68.36 (3)°
*V* = 638.4 (2) Å^3^
*Z* = 2
Mo *K*α radiation
μ = 0.23 mm^−1^
*T* = 293 K
0.1 × 0.1 × 0.1 mm

#### Data collection

Rigaku R-AXIS RAPID diffractometer
Absorption correction: multi-scan (*ABSCOR*; Higashi, 1995 ▶) *T*_min_ = 0.97, *T*_max_ = 0.98
5075 measured reflections
2247 independent reflections
1780 reflections with *I* > 2σ(*I*)
*R*_int_ = 0.018

#### Refinement

*R*[*F* ^2^ > 2σ(*F* ^2^)] = 0.043
*wR*(*F* ^2^) = 0.139
*S* = 1.14
2247 reflections
208 parameters
H-atom parameters constrained
Δρ_max_ = 0.55 e Å^−3^
Δρ_min_ = −0.33 e Å^−3^

### 

Data collection: *RAPID-AUTO* (Rigaku, 1998 ▶); cell refinement: *RAPID-AUTO*; data reduction: *CrystalStructure* (Rigaku/MSC, 2004 ▶); program(s) used to solve structure: *SHELXS97* (Sheldrick, 2008 ▶); program(s) used to refine structure: *SHELXL97* (Sheldrick, 2008 ▶); molecular graphics: *SHELXTL* (Sheldrick, 2008 ▶); software used to prepare material for publication: *SHELXL97*.

## Supplementary Material

Crystal structure: contains datablocks global, I. DOI: 10.1107/S1600536810012870/ds2024sup1.cif

Structure factors: contains datablocks I. DOI: 10.1107/S1600536810012870/ds2024Isup2.hkl

Additional supplementary materials:  crystallographic information; 3D view; checkCIF report

## Figures and Tables

**Table 1:** Hydrogen-bond geometry (Å, °)

*D*—H⋯*A*	*D*—H	H⋯*A*	*D*⋯*A*	*D*—H⋯*A*
O3—H3⋯O2	0.86	1.55	2.410	175
O7—H7⋯O6	0.86	1.55	2.402	175
O9—H9*A*⋯O1^i^	0.85	2.05	2.900	175
O9—H9*B*⋯O1^ii^	0.86	2.11	2.945	161
O10—H10*A*⋯O2^iii^	0.88	2.21	3.024	153
O10—H10*B*⋯O8^iv^	0.88	1.86	2.738	173
O11—H11*A*⋯O4^v^	0.89	2.05	2.917	167
O11—H11*B*⋯O4^iv^	0.88	2.08	2.949	170

Symmetry codes: (i) 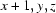; (ii) 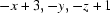; (iii) 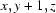; (iv) 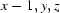; (v) 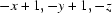.

**Fig. 1.**
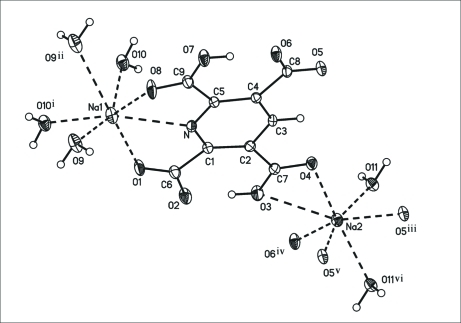
ORTEP view of the title compound, Displacement ellipsoids are drawn at the 30% probability level. [Symmetry codes: (i) 2 - x, 1 - y, 1 - z; (ii) 3 - x, 1 - y, 1 - z; (iii) 2 - x, 1 - y, -z; (iv) x - 1, y - 1, z; (v) x, y - 1, z; (vi) 1 - x, -y, -z.]

**Fig. 2.**
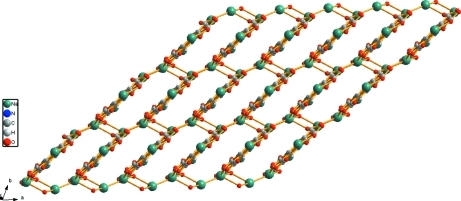
2D layer ∞[Na2(H2pdtc)] parallel to (001) generated from bridging sodium cations and (H2pdtc2-).

**Fig. 3.**
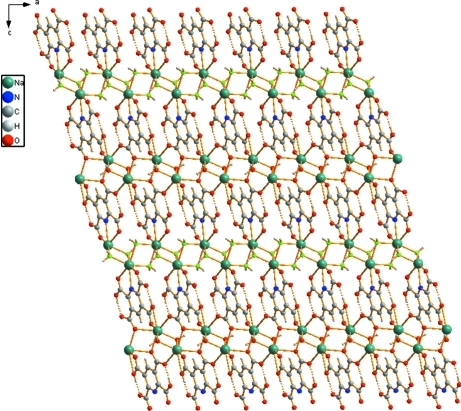
3D framework built from linking ∞[Na2(H2pdtc)] layers by the water molecules (the water molecule linkers are marked in green)

## supplementary crystallographic information

### Comment

A great many metal-polycarboxylate compounds with benzene-1,2,4,5-tetracarboxylic acid (H_4_btec) as ligands have been designed and characterized (Zhang *et al.*, 2010). Analogous in structure to H_4_btec, only four complexes [Ni(H_2_pdtc)(H_2_O)_2_].3H_2_O (Yang *et al.*, 2008), [Zn_4_(pdtc)_2_(phen)_2_(H_2_O)_2_].20H_2_O (Yang *et al.*, 2008), [Cd(H_2_pdtc)(H_2_O)_3_].3H_2_O (Sun, Zhou & Yan, 2009) and [Ni_2_(pdtc)(H_2_O)_3_(2,2'-bpy)].4H_2_O (Sun, Zhou & An, 2009) about pyridine-2,3,5,6-tetracarboxylic acid (H_4_pdtc) have been reported. The four coordination polymers are constructed by linking transition metal centres through the ligands. In this context, we represent a disodium salt of dihydrogen pyridine-2,3,5,6-tetracarboxylate dianion [Na_2_(H_2_pdtc)(H_2_O)_3_].

The asymmetric unit of the title compound consisits of two crystallographically independent Na cations, one dihydrogen pyridine-2,3,5,6-tetracarboxylate dianion (H_2_pdtc^2-^) and three water molecules (Fig. 1). The ligand is deprotonated at 2,5-positioned carboxylate groups. The values of the dihedral angle between the planes of the carboxylic groups and the planar pyridine ring are 3.7 (4)°, 2.0 (5)°, 10.5 (4)°, 2.4 (5)°, respectively. Both Na1 and Na2 ions are seven-coordinated with one N atom, two carboxylate O atoms and four water molecules at Na1 and five carboxylate O atoms and two water molecules at Na2, building highly distorted pentagonal–bipyramidal environment. The average value of the Na—O(water) length [2.494 (2) Å] is closer to the average Na—O (carboxylate) distance [2.498 (2) Å], and both are slightly smaller than the Na—N value [2.654 (2) Å]. The sodium cations are bridged by the (H_2_pdtc^2-^) to generate layers extending infinitely in sheets parallel to (001) (Fig. 2), and further pillared by the water molecule linkers (Fig. 3) to build up 3D framework, which is found to be stabilized by hydrogen bonds from water molecules to carboxylate O atoms (Table 1).

### Experimental

0.0766 g (0.3 mmol) pyridine-2,3,5,6-tetracarboxylic acid and 0.024 g (0.6 mmol) NaOH were successively added to 10.0 ml H_2_O and stirred at room temperature for 2 h, and the resulting colorless solution (pH = 3.48) was then transferred to a 50 ml beaker for slow evaporation at room temperature for several months, affording colorless block crystals (yield: 0.05 g).

### Refinement

H atoms bonded to C atoms were placed in geometrically calculated positions and were refined using a riding model, with *U*_iso_(H) = 1.2*U*_eq_(C). H atoms attached to O atoms were found in a difference Fourier synthesis and were refined using a riding model, with *U*_iso_(H) values set at 1.2*U*eq(O).

### Crystal data

**Table:**

2Na^+^·C_9_H_3_NO_8_^2^^−^·3H_2_O	*Z* = 2
*M**_r_* = 353.15	*F*(000) = 360
Triclinic, *P*1	*D*_x_ = 1.837 Mg m^−^^3^
Hall symbol: -P 1	Mo *K*α radiation, λ = 0.71073 Å
*a* = 5.5844 (11) Å	Cell parameters from 4730 reflections
*b* = 6.6770 (13) Å	θ = 3.3–27.5°
*c* = 18.631 (4) Å	µ = 0.23 mm^−^^1^
α = 81.34 (3)°	*T* = 293 K
β = 86.77 (3)°	Block, colourless
γ = 68.36 (3)°	0.1 × 0.1 × 0.1 mm
*V* = 638.4 (2) Å^3^	

### Data collection

**Table:**

Rigaku R-AXIS RAPID diffractometer	2247 independent reflections
Radiation source: fine-focus sealed tube	1780 reflections with *I* > 2σ(*I*)
graphite	*R*_int_ = 0.018
Detector resolution: 0 pixels mm^-1^	θ_max_ = 25.0°, θ_min_ = 3.3°
ω scans	*h* = −6→6
Absorption correction: multi-scan (*ABSCOR*; Higashi, 1995)	*k* = −7→7
*T*_min_ = 0.97, *T*_max_ = 0.98	*l* = −22→22
5075 measured reflections	

### Refinement

**Table:**

Refinement on *F*^2^	Primary atom site location: structure-invariant direct methods
Least-squares matrix: full	Secondary atom site location: difference Fourier map
*R*[*F*^2^ > 2σ(*F*^2^)] = 0.043	Hydrogen site location: inferred from neighbouring sites
*wR*(*F*^2^) = 0.139	H-atom parameters constrained
*S* = 1.14	*w* = 1/[σ^2^(*F*_o_^2^) + (0.0916*P*)^2^ + 0.0526*P*] where *P* = (*F*_o_^2^ + 2*F*_c_^2^)/3
2247 reflections	(Δ/σ)_max_ < 0.001
208 parameters	Δρ_max_ = 0.55 e Å^−^^3^
0 restraints	Δρ_min_ = −0.33 e Å^−^^3^

### Special details

**Table:**

Geometry. All esds (except the esd in the dihedral angle between two l.s. planes) are estimated using the full covariance matrix. The cell esds are taken into account individually in the estimation of esds in distances, angles and torsion angles; correlations between esds in cell parameters are only used when they are defined by crystal symmetry. An approximate (isotropic) treatment of cell esds is used for estimating esds involving l.s. planes.
Refinement. Refinement of *F*^2^ against ALL reflections. The weighted *R*-factor wR and goodness of fit *S* are based on *F*^2^, conventional *R*-factors *R* are based on *F*, with *F* set to zero for negative *F*^2^. The threshold expression of *F*^2^ > σ(*F*^2^) is used only for calculating *R*-factors(gt) etc. and is not relevant to the choice of reflections for refinement. *R*-factors based on *F*^2^ are statistically about twice as large as those based on *F*, and *R*- factors based on ALL data will be even larger.

### Fractional atomic coordinates and isotropic or equivalent isotropic displacement parameters (Å^2^)

**Table:**

	*x*	*y*	*z*	*U*_iso_*/*U*_eq_	
Na1	1.28244 (19)	0.41119 (18)	0.44136 (5)	0.0360 (3)	
Na2	0.70103 (18)	0.05082 (15)	0.05556 (5)	0.0275 (3)	
N	1.2379 (4)	0.4267 (3)	0.29938 (10)	0.0234 (5)	
C1	1.1039 (4)	0.3240 (4)	0.27451 (12)	0.0222 (5)	
C2	1.0250 (4)	0.3696 (4)	0.20111 (12)	0.0227 (5)	
C3	1.0921 (4)	0.5287 (4)	0.15822 (12)	0.0225 (5)	
H3A	1.0392	0.5653	0.1100	0.027*	
C4	1.2339 (4)	0.6372 (4)	0.18300 (12)	0.0205 (5)	
C5	1.3080 (4)	0.5774 (4)	0.25638 (12)	0.0213 (5)	
C6	1.0527 (5)	0.1626 (4)	0.33584 (13)	0.0280 (6)	
O1	1.1267 (4)	0.1553 (3)	0.39697 (9)	0.0364 (5)	
O2	0.9322 (4)	0.0429 (3)	0.32087 (10)	0.0408 (5)	
C7	0.8762 (5)	0.2689 (4)	0.16184 (13)	0.0252 (5)	
O3	0.7999 (4)	0.1219 (3)	0.19559 (9)	0.0361 (5)	
H3	0.8543	0.0954	0.2396	0.043*	
O4	0.8325 (3)	0.3309 (3)	0.09639 (9)	0.0299 (4)	
C8	1.2832 (4)	0.8074 (4)	0.12553 (12)	0.0231 (5)	
O5	1.1680 (3)	0.8518 (3)	0.06780 (9)	0.0295 (4)	
O6	1.4448 (3)	0.8925 (3)	0.13981 (9)	0.0335 (5)	
C9	1.4680 (5)	0.6635 (4)	0.29836 (13)	0.0270 (6)	
O7	1.5562 (4)	0.8069 (3)	0.26636 (10)	0.0385 (5)	
H7	1.5260	0.8359	0.2205	0.046*	
O8	1.5129 (4)	0.5911 (3)	0.36238 (9)	0.0437 (5)	
O9	1.6891 (4)	0.2384 (3)	0.49492 (11)	0.0466 (5)	
H9A	1.8156	0.2081	0.4655	0.056*	
H9B	1.7251	0.1117	0.5202	0.056*	
O10	0.8582 (4)	0.6942 (3)	0.43369 (10)	0.0363 (5)	
H10A	0.8616	0.8205	0.4131	0.044*	
H10B	0.7414	0.6720	0.4094	0.044*	
O11	0.2943 (3)	0.2647 (3)	0.00399 (9)	0.0304 (4)	
H11A	0.2605	0.3963	−0.0208	0.037*	
H11B	0.1455	0.2949	0.0272	0.037*	

### Atomic displacement parameters (Å^2^)

**Table:**

	*U*^11^	*U*^22^	*U*^33^	*U*^12^	*U*^13^	*U*^23^
Na1	0.0340 (6)	0.0478 (6)	0.0265 (6)	−0.0181 (5)	−0.0043 (4)	0.0044 (4)
Na2	0.0285 (5)	0.0318 (5)	0.0256 (5)	−0.0155 (4)	−0.0043 (4)	−0.0008 (4)
N	0.0272 (10)	0.0255 (10)	0.0180 (10)	−0.0103 (9)	−0.0006 (8)	−0.0027 (8)
C1	0.0233 (11)	0.0233 (12)	0.0200 (12)	−0.0090 (10)	−0.0002 (9)	−0.0012 (9)
C2	0.0238 (11)	0.0224 (11)	0.0227 (12)	−0.0092 (10)	−0.0015 (9)	−0.0036 (9)
C3	0.0277 (12)	0.0245 (12)	0.0150 (11)	−0.0092 (10)	−0.0040 (9)	−0.0018 (9)
C4	0.0223 (11)	0.0206 (11)	0.0190 (11)	−0.0087 (10)	0.0001 (9)	−0.0020 (9)
C5	0.0239 (11)	0.0210 (11)	0.0189 (11)	−0.0087 (10)	0.0008 (9)	−0.0012 (9)
C6	0.0314 (13)	0.0300 (13)	0.0237 (13)	−0.0146 (11)	0.0001 (10)	0.0013 (10)
O1	0.0510 (12)	0.0438 (11)	0.0195 (10)	−0.0264 (10)	−0.0049 (8)	0.0057 (8)
O2	0.0608 (13)	0.0453 (11)	0.0292 (10)	−0.0379 (11)	−0.0054 (9)	0.0056 (8)
C7	0.0299 (13)	0.0243 (12)	0.0228 (13)	−0.0113 (11)	−0.0018 (10)	−0.0031 (10)
O3	0.0517 (12)	0.0412 (10)	0.0275 (10)	−0.0324 (10)	−0.0060 (8)	0.0008 (8)
O4	0.0415 (10)	0.0320 (9)	0.0227 (9)	−0.0210 (8)	−0.0068 (7)	−0.0009 (7)
C8	0.0273 (12)	0.0261 (12)	0.0169 (11)	−0.0116 (10)	0.0006 (9)	−0.0017 (9)
O5	0.0327 (9)	0.0376 (10)	0.0201 (9)	−0.0176 (8)	−0.0049 (7)	0.0046 (7)
O6	0.0455 (11)	0.0461 (11)	0.0213 (9)	−0.0337 (10)	−0.0057 (8)	0.0040 (8)
C9	0.0331 (13)	0.0292 (13)	0.0226 (13)	−0.0171 (11)	−0.0036 (10)	0.0007 (10)
O7	0.0557 (12)	0.0519 (12)	0.0234 (9)	−0.0394 (11)	−0.0088 (8)	0.0027 (8)
O8	0.0642 (14)	0.0651 (13)	0.0189 (10)	−0.0469 (12)	−0.0146 (9)	0.0090 (9)
O9	0.0342 (11)	0.0582 (13)	0.0355 (11)	−0.0105 (10)	0.0001 (9)	0.0132 (10)
O10	0.0387 (10)	0.0452 (11)	0.0304 (10)	−0.0236 (9)	−0.0077 (8)	0.0033 (8)
O11	0.0286 (9)	0.0302 (9)	0.0296 (9)	−0.0098 (8)	0.0007 (7)	0.0019 (7)

### Geometric parameters (Å, °)

**Table:**

Na1—O9	2.337 (2)	C3—H3A	0.9300
Na1—O8	2.379 (2)	C4—C5	1.406 (3)
Na1—O10	2.420 (2)	C4—C8	1.531 (3)
Na1—O1	2.442 (2)	C5—C9	1.528 (3)
Na1—O10^i^	2.498 (2)	C6—O1	1.221 (3)
Na1—N	2.654 (2)	C6—O2	1.287 (3)
Na1—O9^ii^	2.835 (3)	C7—O4	1.235 (3)
Na1—Na1^i^	3.657 (2)	C7—O3	1.278 (3)
Na1—Na1^ii^	3.948 (2)	O3—H3	0.8603
Na2—O11	2.358 (2)	C8—O5	1.225 (3)
Na2—O5^iii^	2.4395 (18)	C8—O6	1.288 (3)
Na2—O6^iv^	2.4477 (19)	O5—Na2^iii^	2.4395 (18)
Na2—O5^v^	2.457 (2)	O5—Na2^viii^	2.457 (2)
Na2—O4	2.4738 (18)	O6—Na2^ix^	2.4477 (19)
Na2—O11^vi^	2.516 (2)	C9—O8	1.221 (3)
Na2—O3	2.840 (2)	C9—O7	1.287 (3)
Na2—C7	3.022 (3)	O7—H7	0.8596
Na2—Na2^vi^	3.444 (2)	O9—Na1^ii^	2.835 (3)
Na2—Na2^vii^	3.724 (2)	O9—H9A	0.8516
N—C1	1.325 (3)	O9—H9B	0.8625
N—C5	1.349 (3)	O10—Na1^i^	2.498 (2)
C1—C2	1.414 (3)	O10—H10A	0.8790
C1—C6	1.539 (3)	O10—H10B	0.8816
C2—C3	1.382 (3)	O11—Na2^vi^	2.516 (2)
C2—C7	1.519 (3)	O11—H11A	0.8872
C3—C4	1.389 (3)	O11—H11B	0.8833
			
O9—Na1—O8	81.23 (8)	O11—Na2—Na2^vii^	120.83 (6)
O9—Na1—O10	151.17 (9)	O5^iii^—Na2—Na2^vii^	40.67 (5)
O8—Na1—O10	99.80 (8)	O6^iv^—Na2—Na2^vii^	146.74 (7)
O9—Na1—O1	112.23 (9)	O5^v^—Na2—Na2^vii^	40.31 (4)
O8—Na1—O1	120.07 (7)	O4—Na2—Na2^vii^	83.00 (6)
O10—Na1—O1	92.37 (7)	O11^vi^—Na2—Na2^vii^	79.10 (6)
O9—Na1—O10^i^	81.74 (8)	O3—Na2—Na2^vii^	108.88 (6)
O8—Na1—O10^i^	150.12 (7)	C7—Na2—Na2^vii^	93.42 (6)
O10—Na1—O10^i^	83.92 (8)	Na2^vi^—Na2—Na2^vii^	102.29 (5)
O1—Na1—O10^i^	89.14 (7)	C1—N—C5	122.15 (19)
O9—Na1—N	119.02 (8)	C1—N—Na1	120.01 (14)
O8—Na1—N	62.02 (6)	C5—N—Na1	116.33 (15)
O10—Na1—N	85.55 (8)	N—C1—C2	121.20 (19)
O1—Na1—N	60.81 (6)	N—C1—C6	110.34 (19)
O10^i^—Na1—N	147.64 (7)	C2—C1—C6	128.5 (2)
O9—Na1—O9^ii^	80.93 (8)	C3—C2—C1	116.0 (2)
O8—Na1—O9^ii^	70.80 (7)	C3—C2—C7	114.6 (2)
O10—Na1—O9^ii^	72.45 (7)	C1—C2—C7	129.44 (19)
O1—Na1—O9^ii^	163.26 (7)	C2—C3—C4	123.8 (2)
O10^i^—Na1—O9^ii^	82.40 (7)	C2—C3—H3A	118.1
N—Na1—O9^ii^	123.02 (7)	C4—C3—H3A	118.1
O9—Na1—Na1^i^	118.79 (7)	C3—C4—C5	116.01 (19)
O8—Na1—Na1^i^	134.81 (8)	C3—C4—C8	114.27 (19)
O10—Na1—Na1^i^	42.77 (5)	C5—C4—C8	129.72 (19)
O1—Na1—Na1^i^	90.98 (6)	N—C5—C4	120.8 (2)
O10^i^—Na1—Na1^i^	41.15 (5)	N—C5—C9	111.11 (19)
N—Na1—Na1^i^	121.70 (7)	C4—C5—C9	128.05 (19)
O9^ii^—Na1—Na1^i^	73.15 (6)	O1—C6—O2	122.8 (2)
O9—Na1—Na1^ii^	45.17 (6)	O1—C6—C1	118.3 (2)
O8—Na1—Na1^ii^	70.94 (5)	O2—C6—C1	118.9 (2)
O10—Na1—Na1^ii^	107.58 (7)	C6—O1—Na1	127.72 (16)
O1—Na1—Na1^ii^	155.73 (7)	O4—C7—O3	120.7 (2)
O10^i^—Na1—Na1^ii^	79.63 (5)	O4—C7—C2	118.50 (19)
N—Na1—Na1^ii^	132.72 (6)	O3—C7—C2	120.8 (2)
O9^ii^—Na1—Na1^ii^	35.76 (5)	O4—C7—Na2	52.54 (11)
Na1^i^—Na1—Na1^ii^	94.41 (5)	O3—C7—Na2	69.52 (13)
O11—Na2—O5^iii^	80.18 (7)	C2—C7—Na2	164.48 (15)
O11—Na2—O6^iv^	82.71 (7)	C7—O3—Na2	85.55 (14)
O5^iii^—Na2—O6^iv^	150.56 (7)	C7—O3—H3	107.5
O11—Na2—O5^v^	161.10 (7)	Na2—O3—H3	160.1
O5^iii^—Na2—O5^v^	80.98 (7)	C7—O4—Na2	104.12 (14)
O6^iv^—Na2—O5^v^	113.75 (7)	O5—C8—O6	123.9 (2)
O11—Na2—O4	101.38 (7)	O5—C8—C4	117.35 (19)
O5^iii^—Na2—O4	89.54 (6)	O6—C8—C4	118.8 (2)
O6^iv^—Na2—O4	117.34 (7)	C8—O5—Na2^iii^	130.76 (15)
O5^v^—Na2—O4	79.82 (7)	C8—O5—Na2^viii^	123.43 (15)
O11—Na2—O11^vi^	90.16 (7)	Na2^iii^—O5—Na2^viii^	99.02 (7)
O5^iii^—Na2—O11^vi^	78.64 (7)	C8—O6—Na2^ix^	128.80 (14)
O6^iv^—Na2—O11^vi^	77.63 (6)	O8—C9—O7	121.0 (2)
O5^v^—Na2—O11^vi^	84.77 (7)	O8—C9—C5	118.5 (2)
O4—Na2—O11^vi^	161.90 (7)	O7—C9—C5	120.5 (2)
O11—Na2—O3	117.19 (7)	C9—O7—H7	112.8
O5^iii^—Na2—O3	134.97 (6)	C9—O8—Na1	126.87 (16)
O6^iv^—Na2—O3	74.36 (6)	Na1—O9—Na1^ii^	99.07 (8)
O5^v^—Na2—O3	77.80 (7)	Na1—O9—H9A	115.5
O4—Na2—O3	47.92 (6)	Na1^ii^—O9—H9A	104.4
O11^vi^—Na2—O3	137.20 (7)	Na1—O9—H9B	118.8
O11—Na2—C7	113.48 (7)	Na1^ii^—O9—H9B	120.3
O5^iii^—Na2—C7	110.79 (7)	H9A—O9—H9B	98.8
O6^iv^—Na2—C7	97.96 (7)	Na1—O10—Na1^i^	96.08 (8)
O5^v^—Na2—C7	74.82 (7)	Na1—O10—H10A	112.2
O4—Na2—C7	23.34 (6)	Na1^i^—O10—H10A	129.4
O11^vi^—Na2—C7	155.41 (7)	Na1—O10—H10B	116.3
O3—Na2—C7	24.93 (6)	Na1^i^—O10—H10B	97.7
O11—Na2—Na2^vi^	46.94 (5)	H10A—O10—H10B	105.2
O5^iii^—Na2—Na2^vi^	74.89 (5)	Na2—O11—Na2^vi^	89.84 (7)
O6^iv^—Na2—Na2^vi^	75.92 (5)	Na2—O11—H11A	122.9
O5^v^—Na2—Na2^vi^	125.55 (7)	Na2^vi^—O11—H11A	121.8
O4—Na2—Na2^vi^	146.06 (7)	Na2—O11—H11B	125.4
O11^vi^—Na2—Na2^vi^	43.23 (5)	Na2^vi^—O11—H11B	100.3
O3—Na2—Na2^vi^	148.10 (6)	H11A—O11—H11B	96.5
C7—Na2—Na2^vi^	159.59 (7)		

Symmetry codes: (i) −*x*+2, −*y*+1, −*z*+1; (ii) −*x*+3, −*y*+1, −*z*+1; (iii) −*x*+2, −*y*+1, −*z*; (iv) *x*−1, *y*−1, *z*; (v) *x*, *y*−1, *z*; (vi) −*x*+1, −*y*, −*z*; (vii) −*x*+2, −*y*, −*z*; (viii) *x*, *y*+1, *z*; (ix) *x*+1, *y*+1, *z*.

### Hydrogen-bond geometry (Å, °)

**Table:**

*D*—H···*A*	*D*—H	H···*A*	*D*···*A*	*D*—H···*A*
O3—H3···O2	0.86	1.55	2.410	175
O7—H7···O6	0.86	1.55	2.402	175
O9—H9A···O1^x^	0.85	2.05	2.900	175
O9—H9B···O1^xi^	0.86	2.11	2.945	161
O10—H10A···O2^viii^	0.88	2.21	3.024	153
O10—H10B···O8^xii^	0.88	1.86	2.738	173
O11—H11A···O4^xiii^	0.89	2.05	2.917	167
O11—H11B···O4^xii^	0.88	2.08	2.949	170

Symmetry codes:  (x) *x*+1, *y*, *z*; (xi) −*x*+3, −*y*, −*z*+1; (viii) *x*, *y*+1, *z*; (xii) *x*−1, *y*, *z*; (xiii) −*x*+1, −*y*+1, −*z*.
